# Age variation in the body coloration of the orb-weaver spider *Alpaida tuonabo* and its implications on foraging

**DOI:** 10.1038/s41598-018-21971-0

**Published:** 2018-02-26

**Authors:** Dumas Gálvez, Yostin Añino, Jorge M. De la O

**Affiliations:** 1 INDICASAT AIP Building 219, City of Knowledge, Clayton, Panama, POBox 0843-01103 Ciudad del Saber, Panama; 2 Programa Centroamericano de Maestría en Entomología, Vicerrectoría de Investigación y Postgrado, Universidad de Panamá, Avenida Simón Bolívar, 0824 Panama; 3 Sistema Nacional de Investigación, Senacyt, Panama; 4 Museo de Invertebrados G. B. Fairchild, Universidad de Panamá, Balboa, Panama; 5 Universidad Nacional, Heredia, Costa Rica

## Abstract

Spiders show a repertoire of strategies to increase their foraging success. In particular, some orb-weaver spiders use attractive body colorations to lure prey. Interestingly, coloration varies with age in many species, which may result in ontogenetic variation of foraging success. By using field observations, laboratory experiments and spectrophotometric analysis, we investigated whether pale juveniles and bright adults of the orb-weaver *Alpaida tuonabo* use different foraging strategies due to ontogenetic variation in coloration. Field observations revealed that foraging success of juveniles and adults was influenced by web properties. However, foraging success increased with body size only in adults, supporting the idea that larger individuals produce a stronger visual signal for prey. The attractiveness of the adult coloration for prey was confirmed in the laboratory with frame-web-choice experiments, in which webs bearing a spider intercepted more bees than empty webs. Our spectrophotometric analysis suggests that the yellow coloration may produce the deceiving signal for prey. Moreover, we identified potential alternative foraging strategies: cryptic juveniles at higher heights and ‘attractive’ adults at lower heights. This study reveals how ontogenetic colour variation may favour the use of alternative foraging strategies in orb-weaver spiders and reduces intraspecific competition.

## Introduction

Spiders exhibit a wide range of foraging behaviours that include active pursuit, sit-and-wait, aggressive mimicry or prey attraction in orb-weaver species through the use of web decorations or body colorations^1^. Interestingly, the strategies employed by spiders can vary with age^2^. For instance, ontogenetic changes may result from exposure to a different type or size of prey^3^ and possibly result in modification of their foraging strategies^4^.

A particular ontogenetic change typical to many orb-weaver spiders is the shift in body coloration from juvenile to adult^5–7^. Surprisingly, the adaptive significance of ontogenetic colour change in animals in general has received little attention^8^. Since body coloration is likely to function for prey attraction in many orb-weaver spiders^9–11^; this difference in coloration may indicate that juveniles and adults occupy different niches, possibly reducing intraspecific competition^12^. Thus, juvenile orb-weavers may rely more on the location or design of the web for foraging while the foraging of adults may be influenced in great part by their coloration.

Overall, experimental work is required to elucidate the evolutionary significance of colour change in animals^13^. Few studies have investigated the ontogenetic variation of foraging strategies in orb-weaver spiders^4,14^ or other group of spiders^15^. For this reason, it is of interest to investigate whether ontogenetic colour variation influences the levels of foraging success or the use of different foraging strategies in orb-weaver spiders.

Here, by using field observations, laboratory experiments and spectrophotometric analysis, we studied the endemic Panamanian orb-weaver *Alpaida tuonabo* Taczanowski, 1878 to determine whether the noticeable yellow-striped adult females differ in their foraging success and strategies as compared to the cryptic pale-red juveniles. For the field observations, we investigated whether the absence or presence of yellow coloration influenced the foraging success of juveniles and adults, respectively. We predicted that: (1) For pale-red juveniles, foraging success is influenced only by properties of the web as capture area, inclination or web height^16^, without the influence of their cryptic coloration, since some insect prey do not seem to locate red colours against a green vegetation background^17^. (2) In yellow-striped adults, together with properties of the web, foraging success is influenced by the size of the spider, based on the idea that the visual signal for prey increases with body size and the innate preference of pollinators for the yellow colour^18^.

Our field observations were contrasted in the laboratory with frame-web-choice experiments for both ontogenetic stages, in which stingless bees were exposed to webs bearing a spider as compared to empty webs. We predicted that webs bearing a spider should intercept more insects only for the adult spiders and no difference between web treatments should be observed for juveniles, as support for our field observations. Since previous studies have shown that is the coloration that attracts insects^11^, we obviated to test the effect of the of the presence of the spider in this experiment.

In addition, using spectrophotometric analysis, we investigated whether the coloration of juvenile and adult *A*. *tuonabo* spiders were visible to a hymenopteran prey over short and long distances, against a vegetation background. For various colour patterns of the spiders, we measured the reflectance spectra in order to estimate chromatic (short-range detection based on colour) and achromatic contrast (long-range detection based on brightness) as compared to the background^19^. This analysis allowed us to test whether juveniles appear cryptic or the adults were noticeable for the average hymenopteran visual system.

## Results

### Field observations

The pale-red juveniles tended to show lower foraging success as compared to the yellow-striped adults (3.4 ± 3.3 vs 4.9 ± 3.9 damaged areas, respectively; GLM of pooled data with ontogenetic stage as only variable: Z = −2.8, P = 0.004). This result can be attributed in part to differences in body size since adult spiders were larger than juveniles (3.3 ± 1.5 vs 2.6 ± 0.7 mm respectively; Wilcoxon test = 641, P = 0.04). Besides, the capture area was not an influential factor, independently of the ontogenetic stage (CA in A in Table 1 for juveniles: 76 ± 42 cm^2^; CA in C in Table 1 for adults: 166 ± 78 cm^2^).

**Table 1:** Full and reduced generalized linear models, with Akaike’s Information Criteria (AIC), used to study foraging success in juveniles (A,B) and adults (C,D) of *Alpaida tuonabo*, showing the significance test for main effects in each model. WH: web height, WI: web inclination, CA: capture area, BS: body size. The reduced models show the coefficient of determination for generalized linear models as proposed by Zhang^48^, which were calculated using the function rsq in R.

Variable	Coefficient	Std. Error	Z	P	
(A) Full model for juveniles					AIC:197
WH	0.008	0.003	2.6	**0.008**	
WI	−0.005	0.008	−0.67	0.5	
CA	0.001	0.002	0.80	0.42	
BS	1.7	1.64	1.03	0.29	
(B) Reduced model for juveniles					AIC:194 R^2^= 0.16
WH	0.007	0.002	2.4	**0.02**	
(C) Full model for adults					AIC:148
WH	0.01	0.002	4.7	**<0.0001**	
WI	−0.01	0.007	−1.8	0.07	
CA	0.0002	0.001	0.19	0.85	
BS	1.2	0.74	1.7	0.09	
(D) Reduced model for adults					AIC:146 R^2^ = 0.70
WH	0.01	0.002	4.7	**<0.0001**	
WI	−0.01	0.006	−1.8	0.07	
BS	1.30	0.65	1.99	**0.046**	

For juveniles, as proposed in prediction 1, only properties of the web influenced foraging success, and were positively correlated with web height (WH in B in Table 1). Neither web inclination (WI in A in Table 1) nor body size were influential (BS in A in Table 1).

For adults, in line with the ‘prey-attraction for body coloration’ hypothesis (prediction 2), foraging success correlated positively with body size (BS in D in Table 1). Moreover, as in juveniles, foraging success also increased with web height (WH in D in Table 1) and was not influenced by web inclination (WI in D in Table 1). Importantly, there is no evidence of collinearity between web height and body size for adults (r = −0.07, t = −0.37, d.f. = 26, P = 0.7), indicating that both variables are not dependent on each other.

The overall negative correlation between body size and web height indicates that adults tend to build lower webs than juveniles (r = −0.24, t = −1.97, d.f. = 61, P = 0.05), suggesting that foraging strategies vary with web height (cryptic coloration at higher heights vs ‘attractive’ coloration at lower heights).

### Laboratory experiments

**Figure 1:**
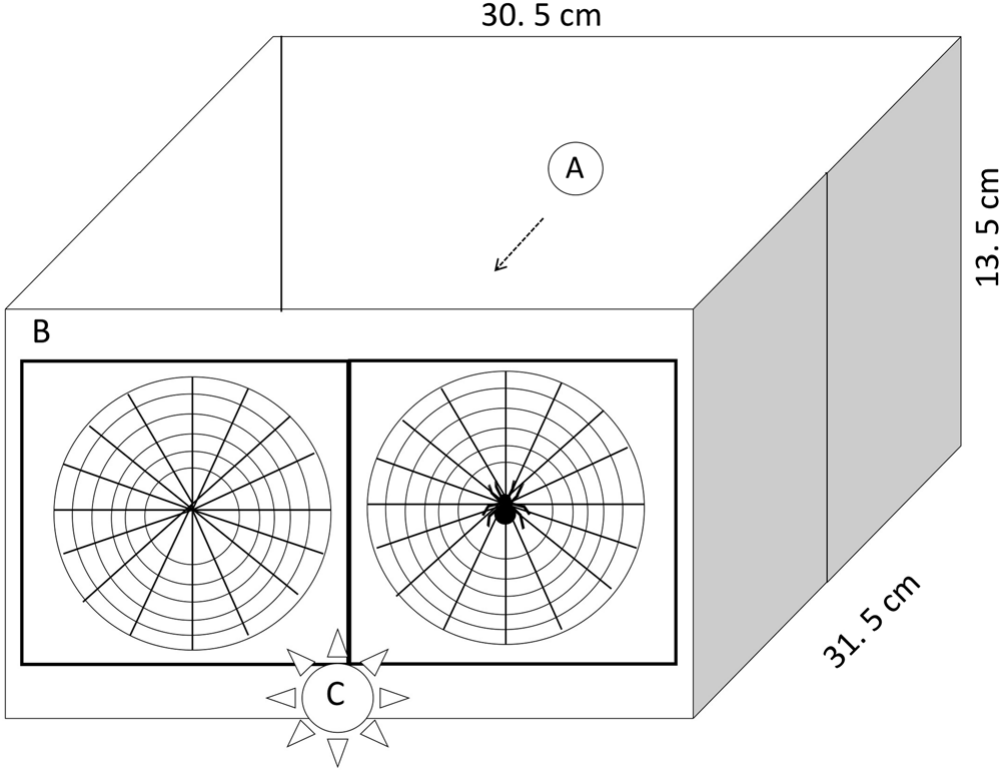
Experimental tunnel in which the stingless bees were exposed to the two web treatments (spider + web: web bearing a spider vs web: web without a spider). The roof of the tunnel is removed in order to reveal the inside. Bees are released in the hole A and bees can try to fly out through one of the frames (B). A light source (L) placed between the two frames mimics daylight reaching from behind the spiders. Despite the position of the light source, this light can reach the dorsum of the spider which always faced the inside of the tunnel.

**Figure 2:**
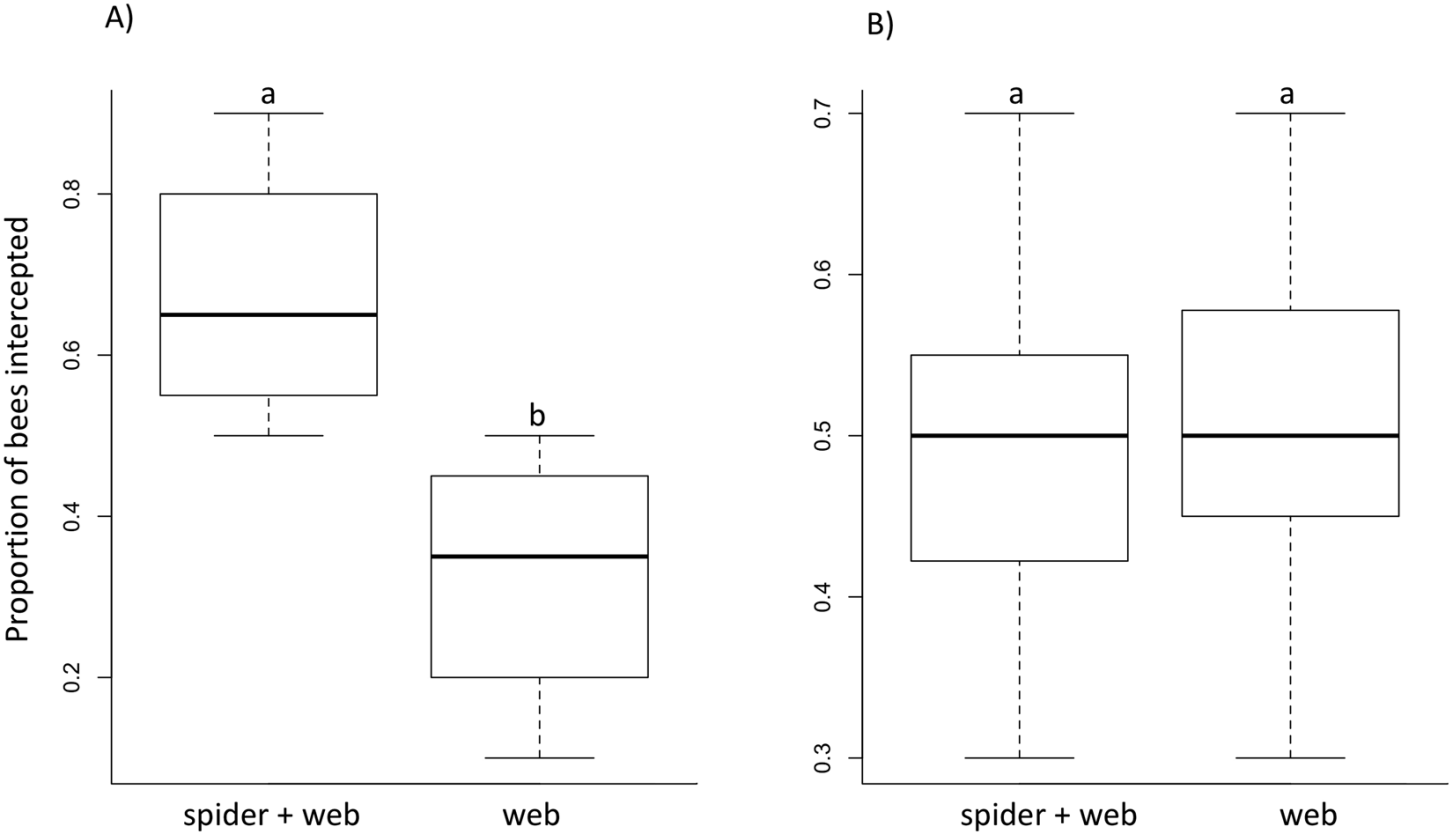
Boxplots of the proportions of bees intercepted in the experimental tunnel for independent trials with adult (**A**, n = 12 spiders, 120 bees) and juvenile spiders (**B**, n = 12 spiders, 118 bees). Each replicate consisted of releasing independently a total of ten bees in the tunnel per pair of frames (spider + web: web bearing a spider, web: web without a spider). Boxes correspond to the interquartile range and solid lines to the median for each group. Different lower cases indicate treatments that differed significantly from one another.

The results from the experimental tunnel (Fig. 1) showed an interaction between the age (adult vs juvenile) and treatment (spider + web vs web, z = 5.1, p < 0.0001). Webs bearing a spider intercepted more bees as compared to the empty web for adults (z = −5.5, p < 0.0001, Fig. 2A), in support of the prediction 2 for our field observations. But no effect was detected for the coloration of juvenile spiders (z = 0.43, p = 0.67, Fig. 2B), in line with prediction 1.

### Spectrophotometric analysis

**Figure 3:**
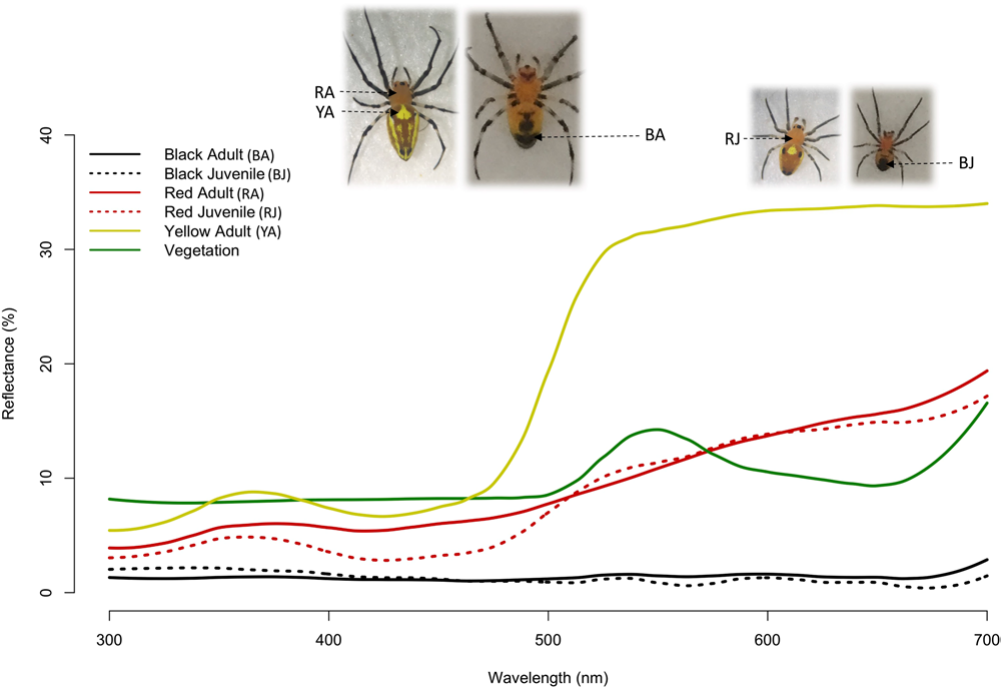
Mean reflectance spectra of different colour patterns in the body of *Alpaida tuonabo* and the vegetation typical to Pipeline Road, Panama. Photographs depict a sample of an adult and a juvenile: the ventral black spots of adults (BA); the ventral black spots of juveniles (BJ); the dorsal red pale colour of adults (RA), the dorsal red pale colour of juveniles (RJ); the dorsal yellow stripes of adults (YA). Older juveniles may have a yellow spot, but most juveniles used in this experiment did not have one. Moreover, we were unable to measure this spot with the spectrometer in the cases in which it was present, due to the small size. For standardization of the measurements of the red colour between the two stages, we measured it in the cephalothorax; which we were unable to measure in the abdomen of adults. This figure was built using the package ‘pavo’ in R^47^.

The visual contrast created by the various body patterns with the vegetation background was similar for both juveniles and adults, with the exception of the yellow stripes in adults that create a distinctive reflectance, with significant contrasts (Table 2 and Fig. 3). This result suggests that a hymenopteran prey is able to see the yellow stripes of adult spiders over short (t = 5.34, d.f. = 7, p = 0.005, Table 2) and long distances (t = 10.2, d.f. = 7, p < 0.0001, Table 1). The red and black colorations may provide camouflage for both adults and juveniles at short distances, since their chromatic contrast values were not significant (Table 2). Besides, the black coloration appears darker than the vegetation over long distances for both adults and juveniles (achromatic contrast, Table 2).

**Table 2:** Summary of one-sample t test for chromatic and achromatic contrast of *Alpaida tuonabo* spiders on a vegetation background from Pipeline Road, as seen by a hymenopteran.

	*Mean* ± *SD*	*t*	*d.f*.	*P*
Hymenoptera vision				
Chromatic				
Adult				
yellow vs background	0.22 ± 0.09	5.34	7	**0.005**
red vs background	0.08 ± 0.04	2.80	9	0.08
black vs background	0.08 ± 0.13	0.77	8	0.5
Juvenile				
red vs background	0.11 ± 0.1	1.64	7	0.42
black vs background	0.10 ± 0.12	1.02	6	0.7
Achromatic				
Adult				
yellow vs background	2.10 ± 0.30	10.2	7	**<0.0001**
red vs background	1.2 ± 0.93	0.61	9	0.55
black vs background	0.24 ± 0.12	−16.9	8	**<0.0001**
Juvenile				
red vs background	1.1 ± 0.93	0.37	7	0.72
black vs background	0.21 ± 0.08	−24.8	6	**<0.0001**

## Discussion

The levels of foraging success varied with the ontogenetic stages of *A*. *tuonabo*, adults showing higher success than juveniles. Web height correlated positively with foraging success for both stages, as observed in other orb-weavers^16^. For juveniles, this is in line with our first prediction that only properties of the web should influence foraging, without the influence of their cryptic body coloration. For adults, foraging success also increased with the spider body size and since the striped pattern covers the whole dorsum, perhaps there is a proportional increase of the ‘signal’ with body size^20^ as support for the ‘prey-attraction for body coloration’ hypothesis^9,10,21^. Therefore, the combined effects of web height and coloration may explain the higher foraging success of adults. Future work is required to disentangle the effects of adult body size and colour patterns on the signal produced for prey.

Interestingly, web height tended to decrease with body size, suggesting that alternative foraging strategies occur during each ontogenetic stage: a cryptic trap at higher heights vs a lure at lower heights. This trend in height differences may allow each ontogenetic stage to exploit different prey and reduce interspecific competition^12,22,23^. Moreover, the coloration variation associated with age and web height may be adaptive against the type of predators encountered during each ontogenetic stage^6,8^.

Our empirical results support the hypothesis that the yellow stripes of the adults improve foraging success, similar to other orb-weaver species with striped patterns^9,10,24^. Although our design does not disentangle the effect of the presence of the adult spider on the web and her coloration, yellow pigmentation has been empirically shown to be visually attractive to diurnal pollinating insects such as hymenopterans^25,26^ and many pollinator insects have an innate preference for yellow^18^. Moreover, the yellow stripes showed a slight peak in the UV range, a light component which is proposed to lure prey insects for many spiders by mimicking food sources or navigation signals^1,27^. It is very unlikely that the adults use pheromone emissions to attract prey, for which the only known cases are found in nocturnal bolas spiders^28^. The cryptic coloration of juveniles does not seem to lure prey to the web and its function remains unknown; perhaps to pass unnoticed by predators or approaching prey^29,30^.

The spectrophotometric analysis also corroborates our field and empirical results, indicating that a hymenopteran is able to see the yellow stripes of adult spiders, at short and long ranges, which possibly deceives the insect to fly towards the web. The lack of significant chromatic and contrast for the dorsal red and ventral black colours indicates that these patterns are not distinguished at short distances by a hymenopteran prey, in line with the idea of a cryptic trap used by juveniles. The black colour in the ventral side of both ontogenetic stages appears less bright at long ranges, however the biological relevance of such pattern for the detection of small size spiders at long range is unlikely^19^.

Why colour production in orb-weaver spiders does not start earlier during development and its ecological significance is intriguing^5^. Body coloration may attract predators^31^, thus we propose that this ontogenetic variation in coloration is adaptive, as it may also increase the survivorship of juveniles by using a cryptic coloration^30^. However, non-adaptive explanations should be considered, for instance, a by-product of physiological processes associated with development or digestion^32^. Moreover, a feasible non-adaptive explanation is the developmental time, space and resources required to acquire the adult coloration^33^.

The striped coloration in adults resembles the patterns of other orb-weaver spiders, which is thought to be the result of an evolutionary trade-off between opposing selective forces, foraging intake and predation risk^24^. This coloration cannot be associated with a negative mechanic stimulus (e.g. spines^34^), since the spider lacks such structures. However, whether the coloration of adults can be associated with an unpalatable taste requires further work, although unpalatability is not common in spiders^5^. This study highlights the effect of ontogenetic colour variation on the foraging strategies used by orb-weaver spiders, a trend that might be frequent across other orb-weaver species that show similar ontogenetic variation in coloration.

## Materials and Methods

### Field observations

We sampled 63 webs of *A*. *tuonabo* along the Pipeline Road in the Soberanía National Park, Panama, containing 35 juveniles and 28 adult females. Juveniles possess dorsal pale-red and ventral black body colorations. Older instars have one or more yellow spots on the thorax but still look cryptic to a human observer; adults possess the red-pale and black coloration plus noticeable yellow stripes that cover most of the dorsal side of the abdomen^35^ (Fig. 3), with little variation across individuals. We did not observe patterns of separate aggregation between the two stages, implying that our results are not the product of differences in microhabitats. For all webs, we measured height, inclination in arc degrees (0° representing a complete vertical web and 90° representing a complete horizontal web) and counted the number of damaged areas in the web as an estimation of foraging success, a standard approximation for foraging success^34,36^.

We measured the vertical diameter (*d*_v_), horizontal diameter (*d*_h_) and height of the free zone (H) for each web. Then we calculated the capture area by using the ellipse-hub formula shown in equation (1)^37^:

1 $$({d}_{v}/2)({d}_{h}/2)\pi -{(H/2)}^{2}\pi $$

Moreover, we estimated body size of the spiders by using body length measurement that were obtained from photographs made in the field and later analysed with the program ImageJ^38^. We used a generalized linear model in R with the function glm (package lme4)^39^, specifying a Poisson regression to test the effects of web height, web inclination, capture area, body size on foraging success. We constructed a full model for each ontogenetic stage with all factors and performed backward selection, removing non-significant factors in order to obtain the best model in terms of Akaike’s Information Criteria (AIC). In order to compare foraging success between ontogenetic stages, we performed a glm with the pooled data using the ontogenetic stage as the only factor. We used a Wilcoxon signed-rank test in order to compare the mean body sizes of juveniles and adults, since the data did not follow a normal distribution. In one case, we performed a Pearson correlation coefficient to test for collinearity between two significant explanatory variables (see D in Table 1).

### Laboratory experiments

To corroborate our field observations on foraging success we carried out a frame-web-choice experiments with the stingless bee *Tetragonisca angustula*, a species that cohabits with *A*. *tuonabo* (pers. obs.) and hymenopterans are an important component of their diet^40^. In an experimental tunnel, similar to other apparatus^41^, bees had to try to fly out through one of two frames bearing webs of similar size at the end of the tunnel (B in Fig. 1), which was facing a dark green background of similar reflectance to the vegetation in the field. Since the black and pale colours are unlikely to lure prey, we performed the experiments only with the dorsum of the spider facing the inside of the tunnel. One frame bore a web with a spider and the other one an empty web. Each bee was kept separately in a tube and introduced into the tunnel through a hole in the opposite end (A in Fig. 1). Bees were not forced to leave the tube and the test began as soon as the bee entered the tunnel. We switched the positions of the frames each time two bees had exited the tunnel or were intercepted in order to eliminate any bias due to frame position (10 bees per replicate, juveniles: n = 12 (118 bees); adults: n = 12 (120 bees). Two of the juvenile replicates consisted of a total of 9 bees since one of the bees was unresponsive for each replicate. We conducted separate experiments for adult and juvenile spiders and the trials were performed after placing the spiders during 20–30 minutes in the refrigerator in order to avoid that the spider moved out of the web. We used a beta regression to analyse the proportion of intercepted bees by each treatment, since the response variable is continuous and limited to the interval 0–1^42^.

### Spectrophotometric Analysis

We measured the reflectance spectra of spiders and vegetation in the laboratory using a fibre optic Avantes UV-VIS-NIR spectrophotometer (model AvaSpec-ULS2048, Avantes BV, Apeldoorn, The Netherlands). We used a xenon light source (200–1000 nm, model AvaLight-XE) as illumination. The reflectance data was collected with the software AvaSoft 8.0 and each measurement was averaged five times by the software. Measurements were taken with the probe at 90° degrees and a white reference and dark calibration were taken before measuring the samples.

We used the reflectance measurement of each sample (different colour patterns and vegetation, Fig. 3) to calculate the receptor excitation values of standard photoreceptors for trichromatic Hymenoptera (UV, blue and green^43^), as described in Théry *et al*.^19^. These equations integrate the receptor sensitivities, the daylight spectrum CIE D65 and the measured reflectance of the sample, which generates the potential proportion of maximum excitation for each of the receptors. From this, the chromatic colour contrast and achromatic contrast between sample and vegetation can be calculated based on the Euclidean distances^19^. For each sample, we obtained the Euclidean distances (chromatic contrast) as compared to the vegetation background using the function deltaS in the package ‘colourvision’ in R^44^, which implements Chittka’s model for trichromatic vision^45^. This chromatic contrast represents the sensitivity of all receptor types of the background substracted from the sample (colour pattern) and it is used for short-range detection. The achromatic contrast (brightness contrast) is used for long-range detection and is obtained by dividing the excitation value of the green receptor elicited by the sample with the corresponding values for the vegetation background^19^. Therefore, a value of 1.0 represents equal brightness between the particular body coloration and the background; a value larger than 1.0 indicates that the spider coloration is brighter than the vegetation and a value lower than 1.0 indicate that it is darker. We used the function Q in the package ‘colourvision’ in R^44^ to obtain the excitation values of the green receptor elicited by the sample and the vegetation background.

We performed one-sample t-tests to compare the chromatic contrasts with the detection thresholds for *Apis mellifera* of 0.05^19^. The achromatic contrasts were compared using the same statistical method with an expected contrast of 1.0 (equal brightness). We performed Bonferroni–Holm corrections for multiple comparisons, and we report adjusted p-values for both chromatic and achromatic analysis^46^.

## Electronic supplementary material

Raw data plots and vision hexagon

reflectance data
