# Supplementary material for: Age variation in the body coloration of the orb-weaver spider Alpaida tuonabo and its implications on foraging
Source: Sci Rep. 2018 Feb 26;8:3599. doi: 10.1038/s41598-018-21971-0 (PMC5827658; doi:10.1038/s41598-018-21971-0)

Supplementary information for the manuscript 'Age variation in the body coloration of the orb-weaver spider Alpaida tuonabo and its implications on foraging'

By Dumas Gálvez, Yostin Añino & Jorge M De la O

The relationship between web height and foraging success expressed as number of damaged areas for juveniles (A) and adults (B) of *Alpaida tuonabo*. The trend line represents the relationship between only the two variables, which was significant when included in the GLM for both cases (A & B).

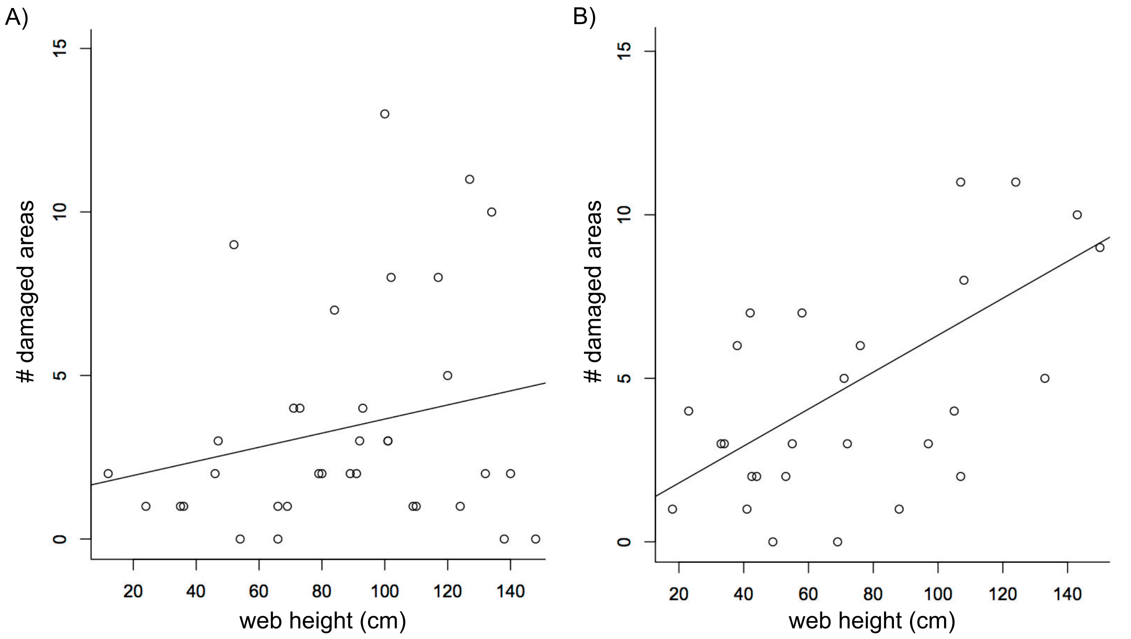

The relationship between body length and foraging success expressed as number of damaged areas for juveniles (A) and adults (B) of *Alpaida tuonabo*. The trend line represents the relationship between only the two variables, which was significant in the GLM for adults (B).

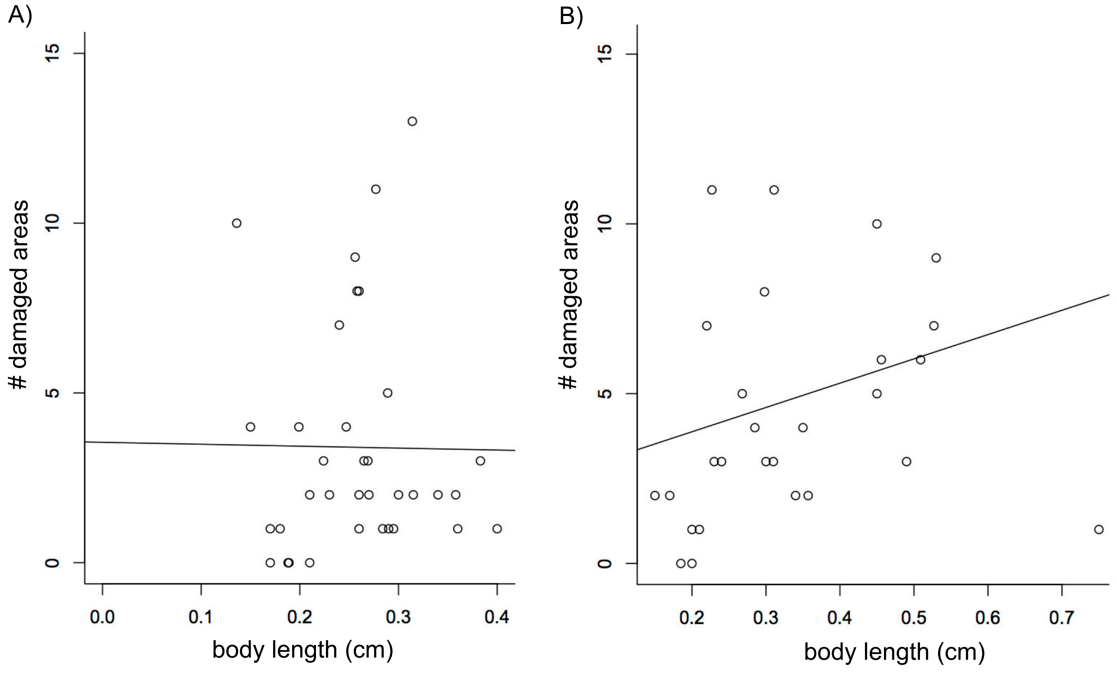

Different color patterns of juveniles and adults of *Alpaida tuonabo* as modeled in the hymenoptera colour hexagon of Chittka[45]. BA: black adult, BJ: black juvenile; RA: red adult; RJ: red juvenile and YA: yellow adult. Hexagon was built using the package ‘colourvision’ in R.

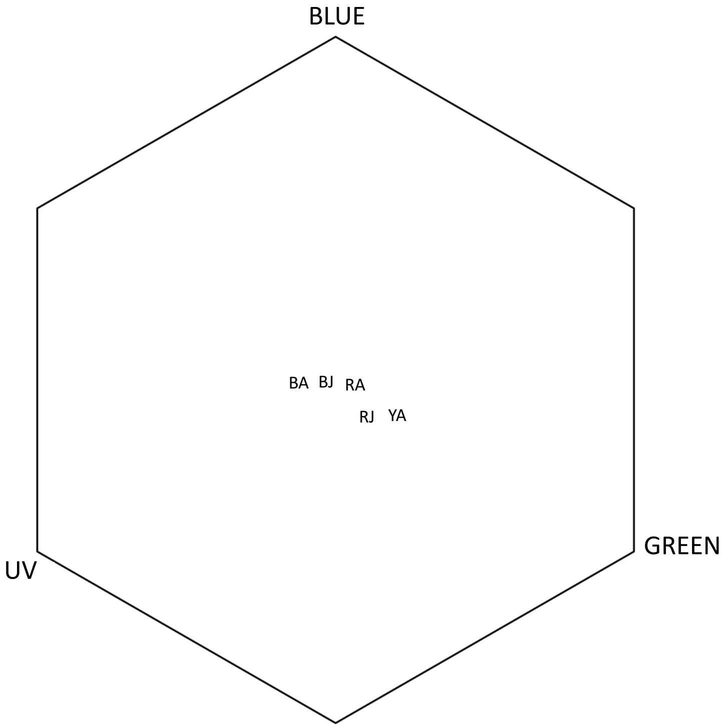

Supplement: Supplementary file 1 — Raw data plots and vision hexagon [file 41598_2018_21971_MOESM1_ESM.pdf]
